# Panache: a web browser-based viewer for linearized pangenomes

**DOI:** 10.1093/bioinformatics/btab688

**Published:** 2021-10-02

**Authors:** Éloi Durant, François Sabot, Matthieu Conte, Mathieu Rouard

**Affiliations:** DIADE, Univ Montpellier, CIRAD, IRD, Montpellier 34830, France; Syngenta Seeds SAS, Saint-Sauveur 31790, France; Bioversity International, Parc Scientifique Agropolis II, Montpellier 34397, France; French Institute of Bioinformatics (IFB)—South Green Bioinformatics Platform, Bioversity, CIRAD, INRAE, IRD, Montpellier 34398, France; DIADE, Univ Montpellier, CIRAD, IRD, Montpellier 34830, France; French Institute of Bioinformatics (IFB)—South Green Bioinformatics Platform, Bioversity, CIRAD, INRAE, IRD, Montpellier 34398, France; Syngenta Seeds SAS, Saint-Sauveur 31790, France; Bioversity International, Parc Scientifique Agropolis II, Montpellier 34397, France; French Institute of Bioinformatics (IFB)—South Green Bioinformatics Platform, Bioversity, CIRAD, INRAE, IRD, Montpellier 34398, France

## Abstract

**Motivation:**

Pangenomics evolved since its first applications on bacteria, extending from the study of genes for a given population to the study of all of its sequences available. While multiple methods are being developed to construct pangenomes in eukaryotic species there is still a gap for efficient and user-friendly visualization tools. Emerging graph representations come with their own challenges, and linearity remains a suitable option for user-friendliness.

**Results:**

We introduce Panache, a tool for the visualization and exploration of linear representations of gene-based and sequence-based pangenomes. It uses a layout similar to genome browsers to display presence absence variations and additional tracks along a linear axis with a pangenomics perspective.

**Availability and implementation:**

Panache is available at github.com/SouthGreenPlatform/panache under the MIT License.

## 1 Introduction

The widespread use of fast and affordable sequencing technologies unveiled how much genomic information was lost when relying on a single and unique reference genome. For instance, it was found that about 10% of additional DNA was not captured by the current human reference genome (Sherman *et al.*, 2019). By leveraging data of multiple references instead, a new era of genomics emerged: Pangenomics. This is now being applied from bacteria to eukaryotes and has been increasingly used in more complex genomes such as humans and plants. As reviewed in Golicz *et al.* (2020), some studies handle this approach through a gene or functional annotation lens while others extend it to DNA sequences, especially when the studied organisms are eukaryotes.

Still, more tools are needed to help pangenomics to reach a broader audience within the scientific community (Computational Pan-Genomics Consortium, 2018; Golicz *et al.*, 2016b; Tranchant‐Dubreuil et al., 2019). While recent progress has been made to compute and store pangenomes (Garrison *et al.*, 2018; Li *et al.*, 2020), the tool landscape is particularly barren when it comes to visualization. Tools such as Pan-Tetris (Hennig *et al.*, 2015), PanViz (Pedersen *et al.*, 2017) or PanX (Ding *et al.*, 2018) were designed for gene-based pangenomes and do not scale well to large-scale eukaryotic studies. Indeed, this gene-centric definition does not take into account positions within genomes, thereby blending paralogs together, and ignoring non-coding sequences despite their crucial influence on phenotypes (Maston *et al.*, 2006). The current trend for sequence-based pangenomes is to use graph visualization software like Bandage (Wick *et al.*, 2015), a general tool for navigating assembly graphs, but alternatives dedicated to pangenomes and their inner properties are yet to be refined and adopted. For instance, Sequence Tube Maps (Beyer *et al.*, 2019) and MoMi-G (Yokoyama *et al.*, 2019) both focus on structural variations from individual genomes but lack information on the pangenome itself. They indeed do not have direct visual cues for the identification of the most represented parts of a pangenome and lose clarity when more than a dozen genomes are involved.

As useful as graph representations may be, they can easily be overloaded with content, resulting in a ‘hairball’ effect that is hard to read and explore (Yoghourdjian *et al.*, 2020). Linear representations of genomes have their own weaknesses (Nielsen and Wong, 2012) but are widely used in a variety of genome browsers, and most efficient when it comes to exploration tasks. Former attempts such as UCSC’s snake tracks (Nguyen *et al.*, 2014) focused on sequence alignments on one reference, which lack clarity when numerous genomes are involved. Here we introduce Panache—the PANgenome Analyzer with CHromosomal Exploration—a web browser-based viewer which renders interactive linear representations of pangenomes as successions of pangenomic blocks, one panchromosome at a time.

## 2 Features

**Fig. 1.**
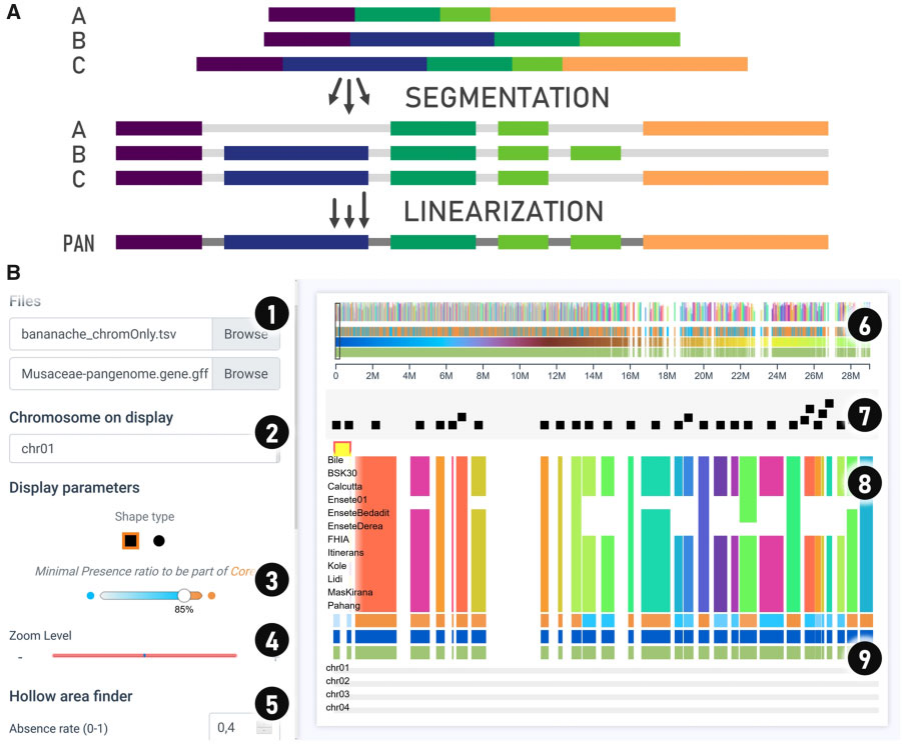
Panache offers a linear representation of pangenomes, with block information detailed through multiple tracks much like classic genome browsers. (**A**) Linearized pangenomes represent chains of present/absent pangenomic blocks on a single string. (**B**) Panache’s interface for browsing through the Presence/Absence matrix and navigating through panchromosomes. The interface is divided into multiple parts: (1) file inputs; (2) panchromosome to display and navigation options; (3) customizable threshold for the core and variable genomes; (4) zoom option to modify blocks’ sizes; (5) further exploration options including a Hollow Area Finder for automatic detection of areas with consecutive absence and sorting options; (6) miniature overview of a whole panchromosome used for navigation on click; (7) track of gene annotations displayed as centered marks in a swarm plot preventing overlaps, detailed cards of annotation are available on hovering; (8) presence/absence matrix of pangenomic blocks, displaying genomes in line and blocks in column; (9) hoverable tracks of summary information (core/variable status, pangenomic coordinates and blocks’ width, amount of repetition and their distribution)

Panache is designed to display a linear representation of pangenomes. This representation is based on the idea that every genome within a pangenome can be divided into multiple blocks, with each block potentially shared with other genomes. Such blocks could be either DNA sequences (extracted from the nodes of a graph pangenome for example) or genes. Blocks from all genomes could be laid out and ordered along a single string, which would then serve as a flattened pan-reference, as illustrated in Figure  1A. One could also imagine ordering the blocks according to an existing genome instead, using its own linear coordinate system as a reference. Every pangenomic block can therefore be represented in an easy-to-browse visualization, with additional tracks of summarized information such as a block’s presence/absence status or whether it belongs to the core genome (most present blocks) or the variable genome (also referred to as dispensable genome).

While a graph representation might give a better sense of structural variations or of a genome’s full sequence within a pangenome, a linear representation allows more reproducibility when exploring data thanks to its fixed and ordered coordinate system. A fixed order allows users to experience the same exploration between visualization sessions, contrary to graphs that may be represented differently every time a file is loaded. Moreover, missing information can be visually hinted at even when not directly available. For example, an additional track can specify which blocks are repeated elsewhere in the pangenome.

The tool comes with a variety of navigation and exploration related functionalities: choosing which panchromosome to display, browsing through it and sorting related individuals with various options based on known phylogenetic information or presence/absence status (gene list or pattern in a selected region). In addition, users can jump automatically to areas enriched in absent blocks with the so-called hollow area finder. It also allows interactive events such as on-the-fly modifications to the core/variable threshold or to the zoom level and hovering over visual elements to display additional information such as functional annotation pop-up windows. All available functionalities are further detailed within Panache’s documentation.

Figure  1B illustrates how Panache displays linear pangenome data using a pangenome generated in banana (Rijzaani *et al.*, 2021). A set of 34 878 genes from 15 genotypes have been grouped into chromosomes and positioned linearly on a panreference. Here, a user can quickly identify lines with missing genes, and which genes belong to the core genome (in orange) or to the variable genome (in blue). Details about the individual gene annotations can be accessed by hovering over the beeswarm-like plot on top of the presence/absence matrix, where genes are represented as non-overlapping marks that can display informative cards of annotation on mouseover.

## 3 Implementation

Panache is a client-side JavaScript web application built with Vue.js 2 and additional libraries, namely D3.js v5 (enabling linkages between data and SVGs) and Vuex. For easy deployment, we have created a Docker container that can run Panache through nginx but the production files are available for deployment through other means.

Panache takes pre-computed pangenome files as input. The main file is a presence/absence matrix file in a BED-like format. Each line stores information about one pangenomic block (either a gene or a sequence) detailed through multiple columns, starting with linear position data and ending with the presence/absence information within every genome. An optional GFF3 file of annotations on the linear pangenomic coordinates may be loaded in addition to the matrix file. The information of genes’ coordinates, exon structure and functional annotations would then be grouped into cards of annotations, available for query on a dedicated track.

As long as the input pangenome file satisfies Panache’s criterias, the pangenome construction method is left up to the users. It is possible to use graph-based pangenomes if they are previously linearized with tools such as BioGraph.jl (https://github.com/nguyetdang/BioGraph.jl). Example files and details about how to format datasets are provided on the GitHub repository.

## 4 Discussion

Panache offers an innovative web interface for the linear representation of pre-computed pangenomes, explorable through a web interface, making the exploration and use of pangenomes easier. Rather than focusing on the nucleotide scale or small variations, Panache intends to work at the genome block resolution (e.g. 1–10 kb), facilitating the discovery of presence absence variation (PAV) patterns across a set of sequenced individuals.

As a lightweight application, it can be embedded easily in an independent manner (e.g. i-frame) to complement other interfaces in existing genome information systems—as illustrated with the Banana genome Hub (Droc *et al.*, 2013). It has already been tested with open access datasets (Golicz *et al.*, 2016a; Rijzaani *et al.*, 2021) and proved to be an effective alternative to existing tools by highlighting inherent pangenomic properties. However, as a visualization tool, results are highly dependent on the methods used to create and linearize the pangenomes. Currently, Panache works efficiently with PAV matrices containing dozens up to about three hundred eukaryotic genomes and will be further improved to deal with larger sample size. Further work on pangenome representations, and particularly on structural variations, is also needed to enhance existing representations.

Current plans for Panache include native support of graph files such as GFA (https://github.com/GFA-spec/GFA-spec) as an input and faster display technologies like WebGL. Panache is still under active development and new features of interest to the community will be added regularly through its GitHub page.
